# Late-Onset Hypoparathyroidism-Induced Hypocalcemia in a Very Low Birth Weight Infant Caused by Undiagnosed Maternal Hyperparathyroidism

**DOI:** 10.1055/a-2837-6826

**Published:** 2026-04-14

**Authors:** Arne Beyer, Meike Franssen, Kai Böckenholt, Jonas Rohde, Ester Domning, Florian Schneider

**Affiliations:** 1 Universitätsklinikum Münster, Pädiatrische Kardiologie, Münster, North Rhine-Westphalia, Deutschland; 2 St. Franziskus-Hospital Münster, Klinik für Kinder- und Jugendmedizin, Münster, North Rhine-Westphalia, Deutschland; 3 Universitätsklinikum Münster, Klinik für Kinder- und Jugendmedizin, Münster, North Rhine-Westphalia, Deutschland

**Keywords:** hypocalcemia, hypoparathyroidism, maternal hyperparathyroidism, parathyroid adenoma, neonatal metabolic disorders, intramuscular vitamin D3

## Abstract

**Objective:**

To describe a rare case of late-onset neonatal hypocalcemia in a very preterm infant caused by maternal adenoma-related hyperparathyroidism (HyperPT) and to discuss diagnostic and therapeutic implications.

**Study Design:**

Case report of a preterm infant born at 29 weeks of gestation with very low birth weight. Clinical course, laboratory findings, maternal history, and management were reviewed to identify the etiology and guide treatment.

**Results:**

The infant developed transient, asymptomatic hypocalcemia during the neonatal period. Maternal evaluation revealed primary HyperPT due to a parathyroid adenoma as the underlying cause. The newborn responded to calcium supplementation and optimized vitamin D therapy.

**Conclusion:**

Maternal HyperPT can cause delayed neonatal hypocalcemia even in preterm infants. Early recognition through coordinated maternal–neonatal evaluation is crucial. Optimal vitamin D administration and close interdisciplinary communication between obstetrics and neonatology are essential for prevention and management.

## Introduction

Neonatal hypocalcemia is diagnosed when ionized calcium falls below 1,0 mmol/L. 1 Ionized calcium is a fraction of total calcium that circulates in the form of Ca ^2+^ -ions and is regarded as the physiologically active form. The sum of ionized calcium, protein-bound calcium, and calcium bound to anions such as phosphate or lactate is called total calcium. Since total calcium values depend on albumin concentration and pH, both of which are often altered in preterm infants, in this case report, only ionized calcium, as a more accurate marker of physiologically available calcium, will be mentioned. 2 3

Neonatal hypocalcemia is classified as early-onset (within the first 3 days of life) or late-onset (typically occurring after 7 days). 4 Possible causes for neonatal hypocalcemia include vitamin D insufficiency, transient hypomagnesemia, acute kidney injury, infections and birth asphyxia, as well as iatrogenic causes: excessive phosphate intake, transfusion of citrated blood or phototherapy for hyperbilirubinemia. 5 Certain maternal conditions can also affect the infant's calcium balance, especially a maternal hyperparathyroidism (HyperPT), which can lead to neonatal hypoparathyroidism (HypoPT). Other causes for neonatal HypoPT are a lack of parathyroid glands, as seen in DiGeorge (22q11 deletion) syndrome, Kearns–Sayre syndrome, or Kenny–Caffey syndrome. 4 5

A little more than thirty cases of neonatal hypocalcemia and HypoPT due to maternal HyperPT have been described since the beginning of the 20th century until today, as Mitsiakos et al have summarized in a systematic overview. 6 Most of them occurred after the first week of life, many of them presented with seizures and/or tetany; only four of the infants in that review were late preterm infants with a minimum of 34 weeks of gestational age, whereas the rest were born term.

Maternal HyperPT during pregnancy is a rare condition and often asymptomatic, making it hard to diagnose. Possible causes are parathyroid adenoma, carcinoma, or hyperplasia. 7 In maternal HyperPT, no matter the cause, high parathyroid hormone (PTH) leads to hypercalcemia via increasing bone demineralization, increasing calcium reuptake in the distal tubule of the kidneys, and increasing intestinal and renal calcium absorption by stimulating the conversion of 25-hydroxy-vitamin-D _3_ (calcidiol) to 1,25-dihydroxy-vitamin-D _3_ (calcitriol) in the kidneys. 8 High maternal calcium levels are transferred to the fetus transplacentally, leading to hypercalcemia in the fetus. 9 Synthesis and secretion of PTH are regulated tightly by serum calcium levels, resulting in a reduced secretion and higher degradation of PTH in hypercalcemia, 10 leading to HypoPT in the infant of a mother with HyperPT. 9 During pregnancy, this does not lead to fetal hypocalcemia, because a constant placental calcium supply is ensured. However, after birth and umbilical cord clamping, when the hitherto constant calcium supply is cut off, this HypoPT can lead to a relevant hypocalcemia in the newborn. 9 The manifestation of the hypocalcemia can vary from an early or more often late onset and can last up to several weeks.

Besides the effect on calcium homeostasis, PTH also increases magnesium reuptake and phosphorus excretion in the kidneys, as well as stimulates conversion of calcidiol to calcitriol, as mentioned above. 8 Those effects lead to characteristic laboratory findings in patients with HypoPT, namely reduced levels of PTH, vitamin D, ionized serum calcium, magnesium, and alkaline phosphatase (AP) as well as an increased level of phosphorus. 11

Calcitriol, on the other hand, the active form of vitamin D, besides increasing intestinal and renal calcium and phosphorus absorption, also promotes bone mineralization. 12 Laboratory results suggestive of a vitamin D deficiency in patients with hypocalcemia as a differential diagnosis of HypoPT are the increased levels of PTH and AP and the reduced level of phosphorus, allowing an effortless distinction from HypoPT. 11

Magnesium plays a dual role in calcium metabolism. While HypoPT can lead to hypomagnesemia due to a decreased renal reuptake, severe hypomagnesemia itself can also lead to HypoPT via a paradoxical suppression of PTH secretion. 13 Hence, hypomagnesemia can be the primary issue, because it can lead to HypoPT, as well as a sign of a secondary outcome, then enhancing the already established HypoPT. Transient hypomagnesemia occurs frequently in neonates, both term and preterm, though it seldom requires substitution. 14

While talking about calcium, one must also consider phosphorus as an important player—alongside its alterations due to the influence of PTH and vitamin D—since high phosphate levels themselves may lead to hypocalcemia; the underlying mechanism is the alteration of calcium-phosphate product and its solubility, leading to increased precipitation and therefore depositions of calcium salt. Causes can be excessive phosphorus intake, which is seen in infants fed with cow's milk or iatrogenic, as well as poor renal excretion, as seen in patients with kidney impairment. 15

The AP is a group of enzymes that, besides many other functions, is characteristically increased in settings with high bone turnover, for example, in vitamin D deficiency, but not in HypoPT. 11 Therefore, it can be used as a differential marker as well.

Neonatal hypocalcemia often lacks clinical symptoms and is therefore diagnosed via electrolyte screening, as seen in this case. The most common clinical features, if they occur, are variable and range from light neuromuscular irritability to severe generalized or focal clonic seizures. 5 Sometimes, signs of poor bone mineralization like cortical thinning, periosteal reactions, or features of rickets can be seen in X-rays, especially in cases of long-lasting HyperPT and vitamin D insufficiency. 16

Preterm infants and those with fetal growth restriction are particularly vulnerable to disruptions of the calcium homeostasis due to multiple factors like hyperphosphatemia, hypomagnesemia, urinary loss of calcium (due to renal insufficiency), and a reduced response to PTH.

Therapeutic options for infants with HypoPT due to maternal HyperPT include calcium supplementation, magnesium substitution when necessary, and vitamin D treatment. Optimizing electrolyte intake while closely monitoring serum and urine levels is crucial. 11

This report highlights the importance of close electrolyte monitoring in preterm infants and the postnatal effects of transplacental transfer, illustrated by a rare case of asymptomatic late-onset hypocalcemia in a very low birth weight (VLBW) preterm infant due to maternal parathyroid adenoma-related HyperPT.

## Maternal History

In our case, a preterm female neonate of 29 weeks' gestational age was born via caesarean section because of maternal thrombocytopenia and hemolysis, as well as a pathological cardiotocograph in the context of an atypical hemolytic uraemic syndrome (aHUS), which in the further course required maternal renal replacement therapy. In a routine assessment 5 months before birth, mild maternal hypercalcemia was found. Control measurements were recommended, to which the patient did not attend. The adherence to a vitamin D therapy and a possible overdose were unclear. Four days prior to birth, the mother was hospitalized again, eventually leading to preterm birth and the diagnosis of aHUS with acute kidney failure. A single course of antenatal steroids with two doses of betamethasone was given 3 days prior to birth. Besides the postpartum diagnosis of aHUS and the hypercalcemia, the mother had no known relevant comorbidities, especially no diagnosis of HyperPT. Shortly after birth, hypercalcemia had intensified to a maximum of 3.09 mmol/L, simultaneously showing severe vitamin D deficiency of 6.8 ng/mL (17 nmol/L) and elevated PTH of 294 pg/mL (31 pmol/L). A connection to the aHUS could not be found. The cause of the maternal HyperPT, a parathyroid adenoma, was diagnosed months later via a parathyroid gland subtraction scintigraphy. The adenoma was eventually surgically resected a few months later.

## Postnatal Development

With a birth weight of 1,235 g (50th percentile), the infant was VLBW with no hint of fetal growth restriction. Clinical examination showed no signs of syndromic features. After birth, there were no complications. Noninvasive ventilation was needed for 13 days. The postnatal weight development was adequate.

We started small enteral feeds with breast milk and diluted formula on the day of birth and added a parenteral nutrition solution with a blend of adequate amino acids, glucose, lipids, electrolytes, vitamins and micronutrients according to the recommendations of the European Society for Paediatric Gastroenterology Hepatology and Nutrition (ESPGHAN) 17 18 19 20 21 22 23 24 25 and adjusted it adequately considering the respective serum electrolytes, weight development and clinical impressions. During the first week of life, enteral feedings and daily fluid intake could be increased daily, and parenteral fluid amounts could accordingly be reduced, reaching full enteral nutrition with around 150 mL/kg/day on the eighth postnatal day. Following the recommendations of the ESPGHAN, we then introduced a breast milk fortifier (Aptamil FMS 4%) to achieve caloric targets and ensure adequate growth.

Following parenteral vitamin D _2_ with 40 IU/kg/day, we furthermore initiated an enteral vitamin D _3_ substitution of 1,000 IU/day (equals 800 IU/kg/day) on the fifth postnatal day as a standard, exceeding ESPGHAN recommendations of 400 to 700 IU/kg/day (maximum 1,000 IU/day), plus the vitamin D _3_ contained in fortified breast milk, which approximately amounts to an additional 670 IU/kg/day. 26

During that period, most electrolytes, like sodium and potassium, showed moderate disturbances that can be marked as normal for a preterm neonate. Ionized serum calcium, though, measured in a point-of-care setting, showed a noticeable drop at the end of the first postnatal week, developing hypocalcemia after the eighth postnatal day with a trough level of 0.7 mmol/L on the 12th postnatal day. Serum phosphorus levels were high with a maximum of 2.7 mmol/L on the 12th postnatal day. Normal values for phosphorus were defined as 1.4 to 2.5 mmol/L. Serum magnesium levels were low with a minimum of 0.53 mmol/L on the 12th postnatal day, with a reference range of 0.63 to 1.05 mmol/L.

Clinical symptoms of hypocalcemia, like muscular irritability, tetany, or seizures, did not occur.

## Investigations and Treatment

Subsequent assessment of intact PTH resulted in a normal value of 27 pg/mL (2.9 pmol/L) with a reference range of 8.5 to 112.3 pg/mL (0.9–11.9 pmol/L). 27 For further evaluation, we measured calcidiol level, which indicated insufficiency with a value of 12 ng/mL (30 nmol/L), almost scratching the cut-off for severe deficiency of below 10 ng/mL (25 nmol/L). 28

At last, urinary electrolytes were measured. Hypocalciuria with a minimum of 0.88 mmol/L and hyperphosphaturia with a maximum of 30.8 mmol/L were found.

Our first approach was to rule out alimentary and therefore iatrogenic causes for hypocalcemia and hyperphosphatemia, so we reviewed parenteral and enteral intakes of those electrolytes plus magnesium.

Parenteral calcium intake in the first week of life consisted of 0.7 mmol/kg/day parenteral calcium solution, which is slightly lower than ESPGHAN recommendations of 0.8 to 2.0 mmol/kg/day parenteral calcium intake, though it has to be seen in the total picture, considering the additional enteral calcium intake via breast milk. 18 Starting with minimal enteral feeds on the day of birth, enteral fluid amounts were increased daily up until a full enteral fluid intake of 150 mL/kg/day was achieved. 29 Enteral calcium intake via breast milk thus reached roughly 1 mmol/kg/day, whilst taking into account that breast milk calcium concentrations vary strongly between individuals, maternal health, and duration of lactation. 30 From our own experience, our VLBW infants, excluding cases like this one, tend to have stable calcium levels with this approach. With the introduction of a milk fortifier described above, enteral calcium intake was elevated to approximately 3.6 mmol/kg/day, meeting ESPGHAN recommendations of 3 to 5 mmol/kg/day ( Table 1 ). 29

**Table 1:** Calcium content of breast milk and breast milk with Aptamil FMS fortifier in absolutes and relative to weight with a daily fluid intake of 150 mL/kg in comparison with ESPGHAN recommendations

Calcium content	mg/dL	mmol/dL	mmol/kg/d
Breast milk (mean) 30	26	0.65	0.98
Breast milk with fortifier (Aptamil FMS 4 g in 100 mL) 26	95	2.4	3.6
ESPGHAN recommendation	–	–	3.0–5.0

Phosphorus intake amounted to 1 mmol/kg/day parenterally, also in accordance with ESPGHAN recommendations of 1 to 2 mmol/kg/day and hitting the targeted calcium/phosphorus ratio of below 1 as well, optimizing bone mineralization and ruling out iatrogenic hyperphosphatemia. 18 As full enteral nutrition with breast milk was achieved as described above, enteral phosphorus intake added up to around 0.72 mmol/kg/day without and 2.4 mmol/kg/day with fortifier, also in agreement with ESPGHAN recommendation of 2.2 to 3.7 mmol/kg/day enteral phosphor intake and achieving an acceptable calcium/phosphor ratio of 1:5, still taking into account the individual variations of breast milk contents ( Table 2 ). 26 29 31

**Table 2:** Phosphorus content of breast milk and breast milk with Aptamil FMS fortifier in absolutes and relative to weight with a daily fluid intake of 150 mL/kg in comparison with ESPGHAN recommendations

Phosphorus content	mg/dL	mmol/dL	mmol/kg/d
Breast milk (mean) 31	15	0.48	0.72
Breast milk with fortifier (Aptamil FMS 4 g in 100 mL) 26	50	1.6	2.4
ESPGHAN recommendation	–	–	2.2–3.7

Parenteral magnesium intake was 0.1 mmol/kg/day, while ESPGHAN recommends 0.1 to 0.2 mmol/kg/day. 18 Enteral intake amounted to approximately 0.2 mmol/kg/day without and 0.48 mmol/kg/day with fortifier, meeting ESPGHAN recommendations of 0.4 to 0.5 mmol/kg/day once more ( Table 3 ). 29 32

**Table 3:** Magnesium content of breast milk and breast milk with Aptamil FMS fortifier in absolutes and relative to weight with a daily fluid intake of 150 mL/kg in comparison with ESPGHAN recommendations

Magnesium content	mg/dL	mmol/dL	mmol/kg/d
Breast milk (mean) 32	3.1	0.13	0.20
Breast milk with fortifier (Aptamil FMS 4 g in 100 mL) 26	7.7	0.32	0.48
ESPGHAN recommendation	–	–	0.4–0.5

Therapeutically, as already described, we began with introducing breast milk fortification to increase calcium intake to a solid level and simultaneously initiated an enteral calcium supplementation. We started with about 0.8 mmol/kg/day enteral calcium substitution, gradually increasing the dose to a maximum of 2.6 mmol/kg/day enteral calcium substitution, as ionized calcium did not rise adequately.

Additionally, we started enteral magnesium substitution, gradually increasing dosage up to a maximum of 0.4 mmol/kg/day. The substitution could be stopped after 18 days as magnesium levels came back to normal. Hypocalcemia persisted, though.

Vitamin D _3_ supplementation was hence elevated to 1,500 IU/day as calcium levels didn't rise properly.

Since high doses of enteral calcium, magnesium, and vitamin D _3_ did not have the desired effect and calcidiol levels were still low, we escalated the therapy to a single intramuscular (i.m.) injection of 5,000 IU vitamin D (equals 3,125 IU/kg), after which we continued enteral vitamin D supplementation with 1,000 IU/day. This therapy regimen eventually led to a rapid response of ionized calcium and even a mild hypercalcemia in just a few days, which was answered with a stepwise reduction of enteral calcium substitution. Additional enteral calcium intake could be abolished at last after 24 days of substitution, when stable normal calcium levels had been accomplished. Vitamin D levels rose to 61 ng/mL (153 nmol/L), which is regarded as safe according to an ESPGHAN position paper from 2013, though well-defined thresholds for vitamin D toxicity have not yet been defined. 28

Until discharge, no further electrolyte supplements were needed. Vitamin D substitution was continued with 1,000 IU/day and a recommendation to proceed with that dosage until a weight of around 3,500 g is reached, then continue with vitamin D substitution of 400 to 500 IU/day following the recommendations of national guidelines but adapting to respective changes in follow-up electrolyte measurements. 33

## Discussion

In this case review, a preterm VLBW infant of 29 weeks' gestational age presented with asymptomatic late-onset hypocalcemia. The most probable cause, because of the maternal history, was a HypoPT due to a maternal adenoma-related HyperPT. Right at the start, the constellation of hypocalcemia with hyperphosphatemia was highly suggestive of HypoPT, as a lack of PTH reduces the resorption of calcium from bone, kidney, and intestine, as well as the renal excretion of phosphorus, as stated above. 8 The low calcidiol level also goes in line with the hypothesis of HypoPT, since stimulation of the conversion of calcidiol to calcitriol is reduced when PTH is deficient. 8 Even though in HypoPT renal calcium uptake is diminished, hypocalciuria as an indicator of hypocalcemia can be present. Hyperphosphaturia can be seen in accordance with hyperphosphatemia. So, in this case, urinary electrolytes did not have any relevant diagnostic value. The only finding not consistent with the suspected diagnosis was the normal value for PTH. This seems paradoxical to the hypothesis, though it at least argues against vitamin D deficiency or high phosphate intake as the primary problem in which PTH would be elevated. This could be explained by a counter-regulation of PTH due to hypocalcemia, leading to a slight increase, or due to physiological variations in PTH levels. A simpler explanation could be the lack of reliable reference ranges for preterm infants of this gestational age, since the reference values of Matejek et al, which were used in this review, were generated out of samples of a rather small number (92) of infants, even though the gestational age and birth weight of these infants are similar to those of the infant in our case. 27

Although HypoPT was our suspected diagnosis, differential diagnoses were investigated. This included the evaluation of enteral and parenteral electrolyte and vitamin D _3_ intake. As described above in detail, calcium, phosphorus, and magnesium intakes were in accordance with ESPGHAN recommendations, ruling out alimentary causes for hypocalcemia. Vitamin D supply was stable, exceeding ESPGHAN recommendations in our approach.

Anyhow, a primary vitamin D deficiency as a cause of hypocalcemia is not likely in this situation because of high phosphorus levels, which would be low in sole vitamin D deficiency. On the other side, the idea of a vitamin D insufficiency as the primary problem is corroborated by the resolution of altered values after i.m. substitution of vitamin D _3_ . In fact, a moderate vitamin D insufficiency might be present after all, not least because of the missing stimulating effect of PTH, aggravating the hypocalcemia, but without being the primary cause.

As we ruled out alimentary causes for hypocalcemia, especially an excessive phosphate intake or a lack of calcium or magnesium, offered a stable vitamin D supply, had no sign of fetal growth restriction, maternal diabetes, asphyxia or infection and saw no clinical features consistent with one of the above listed syndromes that might cause HypoPT, the differential diagnoses left were HypoPT due to maternal HyperPT and transient hypomagnesemia. The double-sided role of hypomagnesemia as both a cause and a consequence of HypoPT cannot be evaluated by a specific test, but the fact that hypocalcemia persisted after magnesium values were normalized via substitution is strongly indicative of hypomagnesemia as a secondary condition. So, since transient hypomagnesemia as a cause of HypoPT is rather a diagnosis of exclusion, and also considering the maternal medical history, the diagnosis of HypoPT due to maternal adenoma-related HyperPT was made, even considering the normal PTH value of the infant.

Our therapy regimen consisted of enteral calcium, magnesium, and vitamin D _3_ substitution in expanding doses followed by an escalation into i.m. vitamin D _3_ . This is a common approach, though not evaluated by controlled studies. The intractability of ionized calcium in our patient despite escalating calcium dosage is most likely explained by the theory that enteral calcium, the bioavailability of which varies anyhow, can only be intestinally resorbed in adequate amounts if vitamin D levels are sufficient. Only then can calcium also be incorporated into the bones. The question of why, in this case, enteral substitution of vitamin D _3_ was insufficient, but i.m. vitamin D _3_ achieved a lasting effect, has also not yet been examined by controlled trials. Literature on the effects of i.m. vitamin D _3_ is scarce in general. A prospective, nonblinded study from China found a significant increase in calcidiol levels 28 days after birth when administering 10,000 IU/kg vitamin D _3_ once to preterm infants under 34 weeks of gestational age in comparison to oral 900 IU/day. 34 Limitations are the small number of patients, the large difference in dosage, and the lack of blinding. Another study including adults showed significantly higher rates of correction of a vitamin D deficiency when giving the same dose of vitamin D _3_ i.m. than when giving it orally. Other relevant studies, especially regarding different ways of vitamin D application in the context of HypoPT in preterm infants, were not found. Possible mechanisms that come to mind could be the higher dosage and/or a superior resorption in the i.m. approach. A reason for that might be a possibly low enteral resorbing capacity when administering vitamin D _3_ orally, especially in preterm infants.

When substituting vitamin D _3_ in a patient with HypoPT, one has to consider that the lack of PTH reduces the conversion of calcidiol to the active calcitriol and therefore also diminishes the effect of substituted vitamin D _3_ . From this perspective, other options that elude this problem might be better. Calcitriol, for example, used as medication, does not need to be enzymatically modified before it unfolds its effect and should thus function well in cases of HypoPT. Other options are alfacalcidole or dihydroachysterole, which contain 1-hydroxy-vitamin-D _3_ , which only has to be hydroxylated once in the liver to calcitriol, therefore evading the renal conversion for which PTH would be required. When one of those medications is chosen, the practitioner has to monitor serum electrolytes extra carefully, as hypercalcemia, as a consequence of rapid resolution of hypocalcemia, might occur. 35 In our case, these preferred options were not available, so vitamin D _3_ was used. That might have led to a longer duration of therapy.

Another therapeutic option comes to mind when talking about HypoPT: a substitution of PTH or an analogue. PTH-based therapies are relatively new and are not yet established in children. Due to the high cost, options like teriparatide (Natpar) or palopegteriparatide (Yorvipath) are used merely in adult patients with chronic HypoPT who do not respond adequately to calcium and vitamin D substitution. 36

In summary, our approach was to rule out common differential diagnoses of hypocalcemia to make the correct diagnosis of HypoPT due to maternal HyperPT and simultaneously start a substitution therapy.

## Conclusion

Hypocalcemia is a common condition in preterm infants. While frequent causes normally resolve quickly after alimentary changes or a short-term substitution therapy, late-onset hypocalcemia in HypoPT due to maternal HyperPT can be intractable and requires longer durations of therapy, as is seen in our presented case. In symptomatic patients, parenteral calcium and possibly magnesium substitution should be started immediately. In asymptomatic patients, as ours, therapy regimen includes mostly enteral substitution of calcium, possibly magnesium, and vitamin D. Calcitriol and other drug options that are independent of the renal conversion of calcidiol to calcitriol, which is diminished in HypoPT, are the preferred regimen for vitamin D substitution, though vitamin D _3_ in high dosage and possibly with i.m. administration can also lead to the desired result, as is seen in our case.

We present a thorough approach to neonatal hypocalcemia, which includes the evaluation of most differential diagnoses. Of course, this can be time-consuming, which is why evaluation for an underlying condition should be limited to those neonates who present with particularly pronounced or intractable hypocalcemia, as is the case in our patient. In the remaining neonates with mild, transient hypocalcemia that are easily managed with alimentary measures or low-dose substitution, evaluation of underlying conditions should be limited to only the bare necessities.

This case is noteworthy, since maternally caused hypocalcemia in preterm infants is a rare condition and standardized diagnostic and therapeutic approaches are missing. Therefore, this case can be sorted into the roughly thirty to forty cases already described above. 6 Our case is particularly valuable in that matter, because changes in electrolytes and vitamin D can be observed thoroughly and in interaction with maternal conditions, thanks to the close laboratory and clinical monitoring that preterm infants receive in the first weeks of life in the clinic. This gives an advantage in analyzing such a case in comparison to out-patient newborns who present with the same condition, only once they get symptomatic, leaving a gap in patient history since diagnostically relevant values are missing.

The case furthermore underlines the need for close electrolyte monitoring in preterm infants, as it can allow early detection of electrolyte disorders before they become symptomatic. In the first days, especially during administration of parenteral nutrition, serum electrolytes, including ionized calcium, should be monitored daily, preferably using microsampling to preserve blood volume. Later on, the frequency of monitoring can be reduced according to the status quo or else adapted to respective changes, for example, in calcium values and initiation of substitution therapy. Mild, transient hypocalcemia in preterm infants that quickly responds to moderate calcium substitution doses might not need further evaluation, whereas intractable, persistent hypocalcemia might be the cause of an underlying condition with a need for treatment. Vitamin D monitoring can be started after 3 to 4 weeks of life according to ESPGHAN, 24 29 but should take place earlier and more frequently in suspected HypoPT.

Moreover, we want to emphasize the need for a close collaboration between connected fields of expertise, in this case, gynecology, neonatology, and adult intensive care doctors, since medical conditions of the mother, like a parathyroid adenoma, might affect the infant. And sometimes, laboratory findings in the neonates can lead to the diagnosis of conditions in their mothers. Raising awareness of that matter and performing close communication can pave the way for quicker and easier diagnoses, as well as an early start of therapy, which might help treat conditions like persistent hypocalcemia before it becomes symptomatic.

For the treatment of HypoPT in preterm infants, especially the dosage, drug selection, and way of administration of vitamin D, further comparative studies are needed, at best leading to the development of national or international guidelines.
